# Branch Retinal Vein Occlusion: Pathogenesis, Visual Prognosis, and Treatment Modalities

**DOI:** 10.1080/02713680701851902

**Published:** 2008-02-21

**Authors:** Jiri Rehak, Matus Rehak

**Affiliations:** Department of Ophthalmology, University Hospital, Palacky University, Olomouc, Czech Republic; Department of Ophthalmology, University of Leipzig, Leipzig, Germany

**Keywords:** branch retinal vein occlusion, pathogenesis, risk factors, treatment, visual prognosis

## Abstract

In branch retinal vein occlusion (BRVO), abnormal arteriovenous crossing with vein compression, degenerative changes of the vessel wall and abnormal hematological factors constitute the primary mechanism of vessel occlusion. In general, BRVO has a good prognosis: 50–60% of eyes are reported to have a final visual acuity (VA) of 20/40 or better even without treatment. One important prognostic factor for final VA appears to be the initial VA. Grid laser photocoagulation is an established treatment for macular edema in a particular group of patients with BRVO, while promising results for this condition are shown by intravitreal application of steroids or new vascular endothelial growth factor inhibitors. Vitrectomy with or without arteriovenous sheathotomy combined with removal of the internal limiting membrane may improve vision in eyes with macular edema which are unresponsive to or ineligible for laser treatment.

## BACKGROUND

### Method of Literature Search

Eligible studies were identified through a comprehensive literature search of electronic databases (Medline, 1966–September 2007 and Science Direct, all years). Additional articles were selected from review of the reference lists of the articles generated from the above search. The following keywords and combinations of these words were used in compiling the search: branch retinal vein occlusion, retinal circulatory disorders, pathogenesis, hematological disorders, risk factors, therapy methods, visual prognosis. In total, 150 of these were used for this mini-review.

### Epidemiology, Classification

Retinal vein occlusion (RVO) is the second most common retinal vascular disorder after diabetic retinopathy and is a significant cause of visual handicap. Its prevalence has been shown to vary from 0.7% to 1.6%.^1^^,^^2^ In a population-based study,^3^ an overall incidence of symptomatic RVO was found in 0.21% of patients aged 40 or older. Hayreh et al.^4^ investigated the demographic characteristics of various types of RVO in 1108 patients (1229 eyes). In this study, a male:female ratio of 1.2:1 was noted in a group of patients with RVO. Of the two main types of RVO, central retinal vein occlusion (CRVO) and branch retinal vein occlusion (BRVO), the latter is more common. A further group is hemi-vein occlusion, a distinct clinical entity presenting as occlusion of only one trunk of the central retinal vein in the area of the anterior part of the optic nerve.^4^ Hayreh et al.^4^ postulated that its pathogenesis is closely related to CRVO.

The first case of BRVO was reported by Leber in 1877.^5^ Some studies showed a higher proportion of BRVO patients older than 65 at the onset of the disease compared to CRVO,^4^^,^^6^ but others found no significance of age in the distribution of CRVO and BRVO.^7^^,^^8^ BRVO is divided into two distinct entities: major BRVO, when one of the major branch retinal veins is occluded, and macular BRVO, when one of the macular venules is occluded. In 66% of eyes with BRVO, there is occlusion of the major branch in the superotemporal quadrant followed by 22–43% of eyes with occlusion of the major branch in the inferotemporal quadrant.^9^ Owing to absent subjective BRVO symptoms in nasal quadrants, the diagnosis of occlusion in this localization is mostly accidental and therefore rare.^10^ Very often BRVO in nasal quadrants is diagnosed only when its complication as bleeding from neovascularizations into the vitreous cavity occurs. The cumulative probability of developing a second episode of occlusion in the other eye within 4 years is about 7% in patients with BRVO.^4^

### Pathogenesis

The pathogenesis of RVO is multifactorial while BRVO may be due to a combination of three primary mechanisms: compression of the vein at the arteriovenous (A/V) crossing, degenerative changes of the vessel wall, and abnormal hematological factors. In the following sections these factors are discussed.

#### Arteriovenous Crossing

Koyanagi in 1928^11^ first reported the association between BRVO and A/V crossing, and now it is established that mechanical narrowing of the venous lumen at these intersections plays a role in the pathogenesis of BRVO. Anatomic features of A/V crossings and secondary effects of arteriolar sclerosis may explain the apparent vulnerability of the crossing site to venous occlusion. In the majority of A/V crossings, the thin-walled vein lies between the more rigid thick-walled artery and the highly cellular retina. The sharing by artery and vein of the common adventitial sheath and the narrowing of the venous lumen that normally occurs at the A/V crossing provide the setting for BRVO.^12^ The risk of occlusion may be accentuated when arteriolar sclerosis results in increased rigidity of the crossing artery. Duker and Brown^13^ provided further support for a mechanical basis of BRVO development when they examined the relative anatomic position of the crossing artery and vein at the site of occlusion in 26 eyes with BRVO. They found in all 26 eyes the artery anterior to the vein (towards the vitreous cavity). Zhao et al.^12^ evaluated the anatomic position of the crossing vessels in 106 eyes with BRVO and found the artery anterior to the vein at the obstructed site in 99% of affected eyes. However, other mentioned risk factors must play a role, too, because in approximately 60% of normal A/V crossings without BRVO the artery lies anterior to vein.^12^

#### Degenerative Changes of Vessel Wall

A number of studies have investigated the histological changes of vessel wall at the A/V crossing.^14^^,^^15^ An investigation by Jefferies et al.^14^ showed that the expected venous compression at the crossing in histological view does not exist. He described the bending of the vein into the nerve fiber layer at this point without its compression. Histological investigation of the venous lumen at the A/V crossing in patients with a number of months to several years duration of BRVO showed organized thrombus with varied extent of recanalization in this part. Seitz^15^ described the clinical histological correlation in one eye with BRVO of a few hours after onset. There was no blood thrombus obliterating the venous lumen at the A/V crossing and even the fundoscopic examination showed strong dilated and tortuous vein distal to the crossing. In the area of the A/V crossing, alteration of the endothelium and intima media was present. Seitz suggests that the trophic changes of venous endothelium and intima media, as they follow the compression from overlaying artery, is the root of the pathogenesis of BRVO.^15^ The formation of the thrombus follows as a secondary process. The findings of Frangieh et al.^16^ support this hypothesis; 90% of the patients in their study had evidence of intima media layer hypertrophy, and all had evidence of intravenous thrombosis.

Systemic hypertension, diabetes mellitus, atherosclerosis, and smoking are reported to be more common in patients with RVO.^1^^,^^2^^,^^10^ Sclerosis of the retinal artery which is associated with these systemic disorders may result in further compression of the vein, when the increased rigidity of arterial wall and contraction of the adventitial sheath shared by artery and vein occur. Mechanical obstruction of the vein through the rigid artery in the A/V crossing may result in turbulent blood flow producing damage to venous endothelium and intima media and the sequence of events leading to occlusion of the vein.^12^^,^^14^ The turbulent blood flow was confirmed by Christoffersen and Larsen in an investigation which analyzed the fluorescein angiograms of 250 patients with BRVO.^17^

#### Hematological Disorders

Some studies have revealed an association between BRVO and hyperviscosity due to high hemotocrit.^18^^,^^19^ Higher blood viscosity increases under conditions of low blood flow and erythrocyte aggregation.^18^ Viscosity is mainly dependent upon the hematocrit (the greater the number of erythrocytes, the larger they aggregate) and plasma fibrinogen (required for aggregation to occur).^20^ Another discussed hematological disorder in the pathogenesis of BRVO is dysregulation of the thrombosis-fibrinolysis balance.^21^ The coagulation cascade including different blood factors results in the production of thrombin which converts circulating fibrinogen to fibrin. The coagulation sequence is held in check and inhibited by specific anticoagulants including protein C, protein S, and antithrombin. Table 1 shows the major disorders studied in patients with RVO. The results of published studies, however, are inconsistent, and the role of coagulation factors in the development of RVO remains unclear.

**TABLE 1:** Most discussed coagulation and anticoagulation disorders in the etiology of BRVO

Resistance to activated protein C (especially factor V Leiden mutation) Protein C or protein S deficiency Deficiency of antithrombin III Genetic mutation in the prothrombin (factor II) gene Anti-phospholipid antibodies Hyperhomocysteinemia

### Resistance to Activated Protein C and Deficiency of Protein C or Protein S

Protein C is serine proteinase whose activated form is a potent inhibitor of coagulation factors V and VIII.^22^ Factors V and VIII are a part of the coagulation cascade leading to conversion of fibrinogen to fibrin. Patients with protein C deficiency frequently manifest superficial and deep venous thrombosis and pulmonary embolism. Protein S and phospholipids are co-factors in the inactivation of factors V and VIII by activated protein C.^22^ An absolute deficiency of protein C or S is relatively rare. Tekeli^23^ and several other authors have reported normal levels in patients with RVO.^24^^–^26 The concept of resistance to activated protein C (so-called APC resistance) was first introduced by Dahlbäck et al. in 1993.^27^ APC resistance was subsequently shown to be a risk factor for venous thrombosis.^28^ More than 90% of patients with APC resistance have been shown to have a single point mutation in factor V gene.^29^ This mutation hinders the degradation of factor V normally occurring through protein C. Several investigators have reported an increased frequency of APC resistance in a cohort of patients with RVO,^30^^–^34 but this association has not been confirmed in other studies.^35^^,^^36^ Moreover, some results are inconclusive due to small patient samples or the lack of control groups. The meta-analysis of Janssen et al.^21^ showed the odds ratios for factor V Leiden mutation in patients with RVO 1.5 (95% CI 0.8–3.2). Despite the evidence of the significance of Leiden mutation, the effect of this hematological disorder in the etiology of RVO is only marginal.^21^

### Deficiency of Antithrombin and Mutation in the Prothrombin Gene

In recent studies of patients with RVO, no significant association with a deficiency of antithrombin or with prothrombin mutation was found.^21^^,^^26^^,^^34^^,^^37^^–^40

### Anti-Phospholipid Antibodies and Hyperhomocysteinemia

Antiphospholipid antibodies (APA) consist of a heterogeneous group of immunoglobulins, mainly anti-cardiolipin antibodies (ACA) and lupus anticoagulants (LA). Circulating APA leads to a hypercoagulable state and recurrent thrombosis through thrombocyte activation and inhibition of the natural anticoagulant pathways by binding of membrane phospholipids. Both the presence of LA and increased level of ACA are associated with a 3- to 10-fold increased risk of venous thrombosis.^41^

An elevated level of the amino acid, homocysteine is now generally accepted to be a risk factor for systemic vascular disease.^42^ Homocysteine appears to have a deleterious effect on vascular endothelium and may induce increased platelet aggregation and thrombosis. Levels of homocysteine may be increased by dietary habits, prescription medicines, or enzymatic mutations affecting homocysteine metabolism.^43^ The results of meta-analyses confirm total homocysteine to be an independent risk factor for RVO.^21^^,^^44^ Loewenstein et al.^45^ investigated the prevalence of genetic mutation in the enzyme methylentetrahydrofolate reductase (MTHFR) whose impaired activity may lead to hyperhomocysteinemia. The prevalence of this mutation was significantly higher in patients with RVO compared with the incidence of MTHFR in a control population. However, these results were not confirmed in other studies.^46^ The meta-analysis of Cahill et al.^44^ showed an association between retinal vascular occlusion and hyperhomocysteinemia but not with the mutation in the gene for MTHFR.

#### Pathogenesis of Macular Edema in BRVO

The development of macular edema (ME) followed by BRVO has been hypothesized to be caused by fluid flux from vessels to tissue according to Starling's law,^47^^,^^48^ which is based on the breakdown of the blood-retinal barrier (BRB) as a result of damage to the tight junctions of capillary endothelial cells,^49^ vitreoretinal adhesion,^50^ and secretion into the vitreous of vasopermeability factors produced in the retina.^51^^,^^52^ Observations by Noma et al.^52^ suggest that in patients with BRVO, vascular occlusion induces the expression of vascular endothelial growth factor (VEGF) and Interleukin-6 (IL-6), resulting in BRB breakdown and increased vascular permeability. Thus, VEGF and IL-6 may contribute to the development and progression of vasogenic ME in BRVO. ME is closely associated with retinal hypoxia, and the degree of hypoxia in the center of the macula corresponds to the decrease in visual acuity (VA). If marked hypoxia persists, irreversible structural changes in the macula occur, and the disturbed VA is almost always lasting. It is generally known that ME and intraretinal hemorrhage occurring in BRVO usually disappear within 6 to 12 months.^53^ In these cases, collateral systems often develop. The main purpose of the treatment is to decrease the duration of edema to prevent photoreceptor damage, if no spontaneous improvement occurs.

### Clinical Signs and Diagnosis

In general, diagnosis of BRVO is not a problem owing to its classical features. Major BRVO can be asymptomatic or with visual blurring usually involving the sector of visual field corresponding to the area of the retina involved. In macular BRVO, there is always a central visual disturbance with normal peripheral vision. Acute BRVO presents characteristic clinical features with flame-shaped, dot and blot hemorrhage, soft and hard exudates, retinal edema, and dilated, tortuous vein in a segmental distribution. Signs of old occlusion are vascular sheathing and venous collaterals. The diagnosis is based on clinical examination under slit lamp and fundoscopy in artificial mydriasis. VA is of great importance for future visual prognosis. BRVO often leads to retinal non-perfusion zones in the occlusion area. Fluorescein angiography is particularly useful in determining the extent of ME and ischemia, although the ischemic areas are often obscured by the presence of intraretinal hemorrhage. Retinal neovascularization occurs in 36% of eyes with an area of non-perfusion greater than 5 disc diameter.^54^

RVO is associated with an increase in vascular causes of death (both cerebral and cardiac) in large prospective follow-up studies.^55^ In all patients with RVO, the systemic risk factors (hypertension, diabetes mellitus, blood lipid disorders) should be investigated and managed by appropriate specialists.

### Natural Course and Visual Prognosis

The visual outcome following the natural course of BRVO is well documented.^56^^–^58 In general, BRVO has a good prognosis: 50–60% of eyes have been reported to have a final VA of 20/40 or better even without any treatment.^56^^–^59 The natural course of BRVO is determined by the site and degree of occlusion, the integrity of arterial perfusion to the affected sector, and the efficiency of the developing collateral circulation.^59^ Chronic ME and bleeding into the vitreous from neovascularizations account most frequently for a poor final VA.^54^^,^^58^^,^^60^ Retinal neovascularization and persistent ME develop in 25% and 60% of eyes, respectively.^57^^,^^61^ Gutman et al.^60^ found that in the natural course of BRVO, only 14% of eyes with chronic ME retained a VA of 20/40 or better, while 86% had a final VA of 20/50 or worse. He concluded that chronic ME has a poor prognosis in terms of final VA.^60^ Schilling et al.^62^ observed a worse visual prognosis in cases of ischemic ME compared to perfused ME. However, findings by Finkelstein^63^ showed that 91% of 23 eyes with macular ischemia recovered vision within one year with a VA of 20/40 or better. The conflicting reports and small number of studied eyes make it difficult to reach definitive conclusions on visual prognosis in patients with BRVO.

VA is a very sensitive indicator of the oxygen situation in the macula. For this reason, pre-treatment VA may be an important prognostic factor. Six studies analyzing the relation between initial and final VA were found.^53^^,^^56^^,^^58^^,^^64^^–^66 Five were used in an analysis of the data of eyes with unsatisfactory final VA (20/200 or worse) in relation to initial VA (Table 2). There were 2 groups; the first consisted of eyes with an initial VA of 20/50 or better and the second group of eyes with an initial VA of 20/200 or worse. In the second group were found a considerably higher percentage of eyes with a final VA of 20/200 or worse, regardless whether the eyes had undergone laser treatment or not. Since there were differently divided subgroups for final VA, the study of Subramanian et al.^65^ was not included in our analysis. Magargal et al.^58^ investigated the visual prognosis in 246 eyes with BRVO divided into two groups: with and without laser treatment. The obtained analysis illustrates that in the group of eyes with an initial VA 20/50 or better, no eye (not receiving laser treatment) and only 13% eyes (had undergone laser treatment) had a final VA of 20/200 or worse, whereas in the group of eyes with an initial VA 20/200 or worse, 83% of eyes (not receiving laser treatment) and 50% of eyes (had undergone laser treatment) had this unsatisfactory final VA. In an analogous way, the data for the final VA 20/50 or better in relation to the initial VA were analyzed (Table 3). We can see that in the group of eyes with an initial VA 20/50 or better, 89% of eyes (not receiving laser treatment), and 75% of eyes (had undergone laser treatment) retained this good VA, whereas in the group of eyes with an initial VA 20/200 or worse, only 14% of eyes (not receiving laser treatment) and only 22% of eyes (had undergone laser treatment) had a final VA 20/50 or better.^58^ Similar data are reported in the other studies (Tables 2 and 3). A chi-squared test with Yates correction was used to analyze the data. In 4 cases, in Table 2, and in 5 cases, in Table 3, respectively, the results were statistically significant (*p* < 0.05). Our analysis shows that in eyes with an initial VA 20/50 or better, the visual prognosis is good even without treatment. It could also be concluded that the cases of BRVO with an initial VA of 20/200 or worse have a statistically significantly poorer visual prognosis than those with an initial VA of 20/50 or better. Subramanian et al.^65^ showed that in patients with BRVO who underwent laser treatment of ME, the level of preoperative VA can be a useful predictor of visual outcome.

**TABLE 2:** Final visual acuity of 20/200 or worse in relation to initial visual acuity. Chi-squared test with Yates correction (*p* < 0.05)

	Initial visual acuity 20/50 or better	Initial visual acuity 20/200 or worse	Chi-squared test *p* < 0.05
Natural course—without laser treatment			
Gutman^56^	5% (1/20)	50% (6/12)	Significant
Magargal^58^	0% (0/35)	83% (24/29)	Significant
Had undergone laser treatment			
Wetzig^53^	25% (2/8)	67% (10/15)	No
Jalkh^64^	0% (0/9)	33% (3/12)	No
Magargal^58^	13% (5/40)	50% (32/64)	Significant
Lang^66^	8% (1/13)	50% (8/16)	Significant

**TABLE 3:** Final visual acuity 20/50 or better in relation to initial visual acuity. Chi-squared test with Yates correction (*p* < 0.05)

	Initial visual acuity 20/50 or better	Initial visual acuity 20/200 or worse	Chi-squared test *p* < 0.05
Natural course—without laser treatment			
Gutman^56^	90% (18/20)	33% (4/12)	Significant
Magargal^58^	89% (31/35)	14% (4/29)	Significant
Had undergone laser treatment			
Wetzig^53^	63% (5/8)	20% (3/15)	No
Jalkh^64^	56% (5/9)	9% (1/12)	Significant
Magargal^58^	75% (30/40)	22% (14/64)	Significant
Lang^66^	77% (10/13)	13% (2/16)	Significant

### Treatment

Current treatment options focus on the sequelae of the occluded venous branch, such as ME, retinal neovascularization, vitreous hemorrhage, and traction retinal detachment. There have been a number of treatment modalities advocated for the management of BRVO (Table 4). Many studies that examine interventions for BRVO suffer from methodological limitations, including insufficient power resulting from small sample sizes, short follow-up periods, absence of a control group or inappropriate control group (absence of placebo or best practice intervention as control groups), and lack of distinction between clinical entities. A number of such investigations have therefore produced conflicting data. Hence, the results of randomized clinical trials are the most important. The complex pathogenesis of this disease requires investigation and treatment of all risk factors (hypertension, diabetes mellitus, blood lipid disorders, hematological disorders).

**TABLE 4:** Treatment modalities for BRVO

Anti-aggregative therapy and firbrinolysis Isovolemic hemodilution Laser treatment Intravitreal and periocular application of steroids Intravitreal injection of VEGF inhibitors Sheathotomy and vitrectomy

#### Anti-Aggregative Therapy and Fibrinolysis

Systemic treatment with oral acetylsalicylic acid, subcutaneous heparin, or intravenous thrombolysis have not been shown to be effective treatments for CRVO, while for BRVO no randomized clinical trials have been published as of the date of this review. Thrombolysis using administration of tissue plasminogen activator intravitreally or directly into the retinal vein (mostly upper temporal branch close to the optic disc) has been demonstrated to improve VA in patients with CRVO,^67^ but the results were based only on noncomparative interventional series. There is no general current acceptance of this treatment.

Houtsmuller et al.,^68^ in a double-blind study, examined the platelet aggregation inhibiting effect of ticlopidine in 54 patients with BRVO less than 3 weeks from the onset of symptoms. Compared with placebo therapy a significant improvement in VA was observed with ticlopidine therapy for six months. In the treated group, 69% of patients experienced an improvement in VA, whereas 52% of placebo group reported improvement.

Troxerutin has been suggested to inhibit erythrocyte and platelet aggregation and to improve erythrocyte deformability, thus reducing blood viscosity and the retinal microcirculation.^69^ A double-blind randomized study of 26 patients with BRVO compared troxerutin with placebo.^69^ At 4 months follow-up, more of the patients receiving troxerutin treatment had a mean VA of 20/40 or better than the control group, although this difference was not found to be statistically significant. After 4 months, all patients were treated with troxerutin for 2 years. At the completion of this follow-up period for those patients initially treated with troxerutin, a significant improvement in VA and improvement of ME was demonstrated. The limitation of this study is that there is no separation in the analysis of results for patients with BRVO and CRVO who were included in the study, too.

Both studies mentioned that investigated the medical treatment of BRVO are limited by a small sample size and short follow-up period (6 and 4 months).

#### Isovolaemic Hemodilution

Chen et al.^20^ demonstrated positive results for isovolemic hemodilution given up to 3 months after the on-set of the symptoms of BRVO in patients with a hematocrit of 35% or more. In this randomized controlled study, 18 patients were treated for 6 weeks with venesection and volume replacement using hydroxyethylstarch and compared to 16 untreated control patients. After a one year follow-up, the final VAs were 20/40 and 20/80 for treated and untreated patients, respectively (*p* = 0.03). Patients with ME and a VA 20/40 or worse underwent 3 months after including into the study macular grid laser photocoagulation (MLG). Sector photocoagulation was applied if ocular neovascularization developed or if, at 3 months, the fluorescein angiogram showed an area of capillary non-perfusion greater than 5 disc areas. 28% of the hemodiluted patients required MLG compared to 44% of the control group; this difference was not statistically significant (*p* = 0.2). Sector photocoagulation was required by 50% of both groups of patients.^20^

Hydroxyethylstarch has a capacity to expand the plasma volume by up to 172% of the volume infused and has a duration of action of approximately 36 hours.^70^ It is non-antigenic and has a low incidence of allergic reactions.^71^ Poupard et al.^72^ randomized 25 patients to either hemodilution with dextran for 21 days (*n* = 10), hemodilution combined with heparin for 21 days (*n* = 10), or heparin treatment for 21 days followed by anti-vitamin K drugs for a further 30 days (*n* = 5). The study showed that, for those receiving heparin followed by anti-vitamin K drugs, mean VA remained unchanged to baseline values by 60 days. For those treated with hemodilution and heparin, a statistically significant increase in VA was found by 60 days. For those treated with hemodilution alone, a significant improvement in VA was found by day 14. In a randomized study by Hansen et al.^73^ of 35 patients with BRVO, 18 patients were treated by hemodilution for a period of 5 to 6 weeks (targeted hematocrit 30–35%). A control group of 17 patients were only observed. At follow-up 12 months later, 25 patients had completed the therapy. Seven of the 13 who received hemodilution demonstrated a VA increase of 2 lines or more compared with none of the 12 patients who did not receive hemodilution (*p* < 0.005). Reported complications of hemodilution include headache, exertional dyspnea, tiredness, deep vein thrombosis, and hypotension. The treatment was noted to be generally well-tolerated even in elderly patients.^20^^,^^73^^,^^74^

The use of hemodilution to treat BRVO is currently not generally accepted. Interpretation of the above-mentioned studies is difficult because most of them incorporated other treatments in combination with the hemodilution. Further prospective randomized trials with adequate controls and sufficient follow-up are required for any definitive conclusions and recommendations.

#### Arteriovenous Crossing Sheathotomy and Vitrectomy

Osterloh and Charles^75^ first reported improvement in VA in patients with BRVO after treatment using the technique of surgical sheathotomy. The principle steps of this procedure are a pars plana vitrectomy followed by separation of the retinal artery from the vein by creating an incision in the adventitial sheath adjacent to the A/V crossing and then separation of the adhesions. Several studies have shown significantly better functional outcomes in patients treated by sheathotomy compared to controls (Table 5).^75^^–^96 Reported complications are few but include cataract, hemorrhage, retinal tears, postoperative gliosis, and retinal detachment.^75^^–^96 Garcia-Arumi^76^ described a combination of A/V sheathotomy and injection of thrombolytic into the occluded vein which resulted in thrombus release in 28% cases and significant correlation with early surgery and better final VA. The role of the sheathotomy alone in visual improvement is insufficiently clear. Some authors suggest that vitrectomy is the most important part of the sheathotomy surgery, leading to reduction of ME.^77^^,^^80^^,^^96^ Yamamoto et al.^77^ compared the effect of sheathotomy combined with vitrectomy to the effect of vitrectomy alone and found no advantage of sheathotomy. Eyes with pre-existing posterior vitreous detachment were not studied. For this reason, the benefit of vitrectomy of these eyes is unknown. Surgical detachment of posterior hyaloid could be more important than the sheathotomy itself .^78^ The vitreous is postulated to have a role in the pathogenesis of neovascularization and ME, which may complicate BRVO and its removal may help in the management of these sight threatening complications.^78^ Vitrectomy and removal of the posterior hyaloid with peeling of the internal limiting membrane (ILM) appears to improve oxygenation of the retina, which may lead to visual improvement.^97^^,^^98^ Peeling of the ILM improves the surgical outcome during A/V adventitial sheathotomy, too.^84^ To date, no randomized clinical trials on the surgical treatment of BRVO have been published. Any evidence supporting these procedures is based on non-ramdomized case series only.

**TABLE 5:** Summary of studies evaluating the treatment of macular edema in BRVO by sheathotomy (VA = visual acuity, ME = macular edema, ILM = internal limiting membrane)

Author	Study type	Patients	Follow-up (mean)	Outcomes	Comments
Osterloh and Charles^75^	Case report.	1 eye.	8 months.	VA improved from 20/200 to 20/25.	First report of sheathotomy.
Garcia-Arumi et al.^76^	Prospective interventional nonrandomized study.	40 eyes—all underwent vitrectomy, sheathotomy and injection of 25 mg of tissue plasminogen activator into occluded vein.	13 months.	Thrombus release in 11 eyes (27.5%)—correlated with early surgery. VA increased from 20/100 to 20/40 (p = 0.016).	
Yamamoto et al.^77^	Retrospective interventional comparative case series.	20 eyes—sheathotomy 16 control eyes (posterior vitreous detachment via vitrectomy).	12 months.	VA: significantly better in both groups (p = 0.008 and p = 0.001, respectively). VA and foveal thickness were not significantly different between the groups.	
Charbonnel et al.^78^	Prospective nonrandomized, interventional case series.	13 eyes—sheathotomy.	7 months.	Improvement in VA ≥ 2 ETDRS lines in 9 eyes (69%).	Absence of previous posterior vitreous detachment correlated with improvement in VA.
Sohn et al.^79^	Retrospective interventional case series.	22 eyes—sheathotomy + ILM peeling in all eyes.	3 months.	Improvement in VA (log MAR) from 0.79 ± 0.29 to 0.57 ± 0.33 (p < 0.01).	All eyes pretreated with grid laser or triamcinolone.
Kumagai et al.^80^	Prospective, randomized, comparative, interventional study.	Group 1: 18 eyes—sheathotomy. Group 2: 18 controls (vitrectomy without sheathotomy).	31 months.	VA (log MAR) in group 1: 0.52 → 0.08. In group 2: 0.53 → 0.014. Differences between group 1 and 2 was not significant.	
Avci et al.^81^	Retrospective interventional comparative case series.	11 eyes—sheathotomy. 10 control eyes—grid laser photocoagulation.	9 months.	VA (log MAR): sheathotomy: 0.84 → 0.36. Grid laser: 1.06 → 0.82. Difference was significant.	
Horio et al.^82^	Interventional case series.	7 eyes.	6 months.	Significant improvement in retinal blood flow (p < 0.01) and reduced macular thickness (p = 0.03).	
Lakhanpal et al.^83^	Retrospective interventional case series.	12 eyes.	49.9 weeks.	VA (logMAR) improved from 1.00 ± 0.32 to 0.56 ± 0.28 (p = 0.0003).	25-gauge transvitreal limited arteriovenous crossing manipulation without vitrectomy.
Mester et al.^84^	Prospective interventional nonrandomized case-control study.	43 eyes—sheathotomy. 16 eyes additionally + ILM peeling. 25 control eyes.	6 weeks.	26 patients (60%) gained ≥ 2 lines of VA. Better result in patients with ILM peeling. ME and intraretinal hemorrhage resorbed in all patients.	All patients had isovolaemic hemodilution for 10 days.
Opremcak et al.^85^	Prospective interventional case series.	15 eyes.	6.5 years.	Snellen VA improved in 10 patients (67%) by an average of 4 lines vision (range 1–9 lines). In 3 patients resolution of ME but no improvement of VA.	Retinal vascular bleeding in 2 patients.
Asensio Sanchez et al.^86^	Prospective interventional nonrandomized study.	13 eyes—sheathotomy, 5 eyes underwent additionally ILM peeling.	12 months.	VA improved in 12 patients (92%). Better results in patients with ILM peeling.	
Lerche et al.^87^	Prospective nonrandomized intervention case series.	12 eyes – sheathotomy.	3 months.	VA (logMAR) improved from 0.74 to 0.56.	
Mason et al.^88^	Prospective, nonrandomized, comparative interventional study with concurrent control group.	20 eyes—sheathotomy. 20 control eyes (10 of them without intervention and another 10 underwent grid laser).	14 months (sheathotomy). 19 months (controls).	VA improvement: Sheathotomy: from 20/250 to 20/63. Controls: from 20/180 to 20/125 (p = 0.02). 45% of the surgical group had final VA ≥ 20/40 compared with 15% of the controls.	Data only for whole control group together.
Cahil et al.^89^	Retrospective non-controlled case series.	27 eyes—sheathotomy.	12 months.	Resolution of ME in 8 (29.6%) patients, reduction in 14 (51.8%) and persistence in 5 (18.5.%).	
Becquet et al.^90^	Prospective nonrandomized interventional case series.	6 eyes (sheathotomy + ILM peeling. 6 controls (ILM peeling only).	6 months.	Significant improvement of VA in both groups. No difference in VA or foveolar thickness between the groups (p = 0.5; p = 0.6 respectively).	
Martinez-Soroa et al.^91^	Retrospective interventional case series.	17 eyes—sheathotomy.	6 months.	Improvement in VA from 0.26 to 0.4. 53% patients improved ≥ 4 lines (Snellen).	
Le Rouic^92^	Retrospective interventional case series.	3 eyes—sheathotomy.	10 months.	No improvement in VA observed.	All patients with initial VA < 20/40.
Dotrelova et al.^93^	Retrospective interventional case series.	3 eyes—sheathotomy.	12 months.	VA improved in 2 patients to 20/40, in 1 patient stabilized (20/180).	
Shah et al.^94^	Retrospective interventional case series.	5 eyes—sheathotomy.	6.5 years.	VA preoperative in all patients ≤ 20/200. Improved in 4 eyes from 20/30 to 20/70. 1 eye with counting fingers remained unchanged.	
Crafoord et al.^95^	Retrospective interventional case series.	12 eyes—sheathotomy.	20 months.	VA improved in 9 eyes (75%), in 1 eye (8.3%) remained unchanged and deteriored in 2 eyes (16.7%).	2 patients received additionally 25 mg triamcinolone acetonide at the end of the surgery.
Han et al.^96^	Retrospective interventional case series.	20 eyes—pars plana vitrectomy and dissection of the arteriovenous crossing without separation of the vessels.	10.5 months.	In 16 eyes (80%) improved VA ≥ 2 lines. Mean improvement of VA (logMAR) was = 0.44 ± 0.14 (p = 0.016).	

#### Steroids

##### Intravitreal Corticosteroids

In several nonrandomized comparative studies, intravitreal corticosteroids were successfully used for the treatment of BRVO. Currently published randomized studies are very rare and limited by virtue of evaluating patients with ME of different etiology, making comparisons difficult. In various studied doses from 4 to 25 mg, triamcinolone acetonide (TA) has been reported to be effective^99^^–^117 (Table 6). In a randomized, interventional, three-arm clinical trial, Avitable et al.^99^ compared the results of treating diabetic patients and a small group of BVRO patients with cystoid ME by TA and MLG. From a total of 63 patients, 22 were treated by TA (4 mg), 21 underwent MLG, and in 20 patients these methods were combined (TA + MLG). The greatest improvement in VA was found in patients treated by TA combined with MLG. VA (log MAR) in this group increased significantly from 0.83 at baseline to 0.20 at the end of follow-up 9 months later (*p* = 0.003). In patients treated by TA, VA improved significantly, from 0.82 at baseline to 0.23 at 9 months after injection (*p* = 0.04). VA in the group of patients treated by MLG remained the same. The results of this study are limited, however, owing to the different ME etiologies in evaluated patients; only 6 patients had ME secondary to BRVO. Oh et al.^100^ used a retrospective interventional case series to compare VA after single TA injection (4 mg) in 10 patients with mean duration of ME ≤ 3 months after onset of BRVO versus 10 patients with ME > 3 months after onset. In patients with a disease duration ≤3 months, VA significantly improved from baseline over 6 months of follow-up. However in those with a duration of >3 months, improved VA, though apparent at one month, was not maintained at 3 or 6 months after TA injection. This study is limited by its retrospective design and short follow-up period. Ozkiris et al.^101^ evaluated the effect of TA injection on persistent ME in BRVO that failed to respond to previous laser photocoagulation. During a mean follow-up time of 6.2 months, best corrected VA (log MAR) improved significantly (*p* < 0.001) from 1.01 at baseline to 0.55 at one month after the injection. VA after 3 months was 0.56, and at the end of follow-up was 0.62. The authors concluded that intravitreal application of TA may be helpful in patients who do not respond to laser photocoagulation. However, in published studies, the resulting reduced macular thickness and improved VA, is only temporary and requires repeated treatment. One to four times re-application has been reported. Cekic et al.^103^ performed a retrospective chart review of 13 patients who underwent intravitreal injections with 4 mg TA. Six eyes received a single injection. Repeated injections were performed in 1 eye twice, 4 eyes three times, and 2 eyes four times. During a mean follow-up of 13 months, central foveal thickness decreased by more than 50%. Final VA improved in 7 eyes (range 2–6 Snellen lines), remained the same in 4 eyes (range 0–1 Snellen lines), and worsened in 2 eyes (range 1–4 Snellen lines) compared to baseline. Retinal thickness decreased in all cases, while vision improved in most cases. One of the most common side effects of TA was steroid-induced elevation of intraocular pressure.^118^ Other complications were infectious endophthalmitis, post-injection steroid-induced cataract, and retinal detachment.^119^^,^^120^ Reported risk of infectious endophthalmitis per injection range was from 0.1% to 1.6%.^120^ The most recent report by Bhavsar et al.^121^ found in two large studies-Diabetic Retinopathy Clinical Research Network (DRCR.net) and SCORE (Standard Care versus Corticosteroid for Retinal Vein Occlusion), an endophthalmitis prevalence of 0.05% (one case in the 2009 injections).

**TABLE 6:** Summary of studies evaluating the treatment of macular edema in BRVO by intravitreal application of triamcinolone acetonide (TA = triamcinolone acetonide, VA = visual acuity, ME = macular edema, MLG = macular laser grid photocoagulation)

Author	Study type	Patients	Follow-up (mean)	Outcomes	Comments
Avitabile et al.^99^	Randomized interventional, parallel, three-arm clinical trial.	Intravitreal TA (4mg): 22 eyes. MLG: 21 eyes. TA+MLG: 20 eyes.	9 months.	TA group: VA improved from 0.82 to 0.23 log MAR (p = 0.04). MLG-group: VA unchanged. TA+MLG group, VA improved from 0.83 to 0.20. logMAR (p = 0.003).	Different etiology of ME, only 6 eyes with BRVO.
Oh et al.^100^	Retrospective interventional comparative case series.	20 eyes with ME (4 mg TA) Disease duration: 10 eyes ≤ 3 months; 10 eyes > 3 months.	6 months.	Group ≤ 3 months: VA (logMAR) improved from 1.07to 0.63 in 1 month (p = 0.012) and to 0.34 in 6 months (p = 0.005).	
Ozkiris et al.^101^	Retrospective, non-controlled case series.	19 treated eyes (8 mg TA).	6.2 months.	VA (logMAR) improved from 1.01 ± 0.16 to 0.62 ± 0.22. VA improved in 17 eyes and remained unchanged in 2 eyes.	
Jonas et al.^102^	Prospective nonrandomized comparative study.	10 treated eyes (20 mg TA). 20 untreated controls.	TA patients: 10.1 months. Controls: 6 months.	TA patients: VA increased from 0.27 ± 0.11 to 0.45 ± 0.27 (p = 0.02). Controls: VA decreased significantly (p = 0.007).	VA increased higher in non-ischemic group. Significant increase of intraocular pressure in treated group.
Cekic et al.^103^	Retrospective non-controlled case series.	13 eyes (4 mg TA).	13 months.	VA: improved in 7 eyes, remained the same in 4 eyes, worsened in 2 eyes. Foveolar thickness decreased in 56% of patients (p < 0.001).	VA improvement significantly correlated with patient age (p = 0.026).
Lee et al.^104^	Retrospective, non-controlled case series.	6 eyes (4 mg TA).	149.5 days.	Improvement in VA ≥ 2 lines in 5 eyes (83.3%). VA from 20/166 to final 20/106.	3 eyes treated with re-application of TA.
Ozkiris et al.^105^	Retrospective interventional comparative case series.	15 eyes (8 mg TA). 19 eyes MLG.	6.3 months.	VA (logMAR) improved in TA group from 0.98 to 0.24 and in MLG group from 1.02 to 0.5 (in both groups p < 0.001). Improvement in TA group was significantly higher than in MLG (p < 0.001).	
Yepremyan et al.^106^	Retrospective, non-controlled case series.	12 eyes (4 mg TA).	15.3 months.	VA improved >3 lines in 50% of eyes after 1 month and in 42% of eyes at last follow up.	8 eyes developed recurrent ME at an average of 5.5 months after initial TA injection.
Cheng et al.^107^	Prospective nonrandomized interventional comparative study.	16 eyes (4 mg TA). 11 controls (without TA).	103 days in TA-group. 94.5 days in controls.	VA (logMAR). In TA-group: improvement from 0.77 ± 0.43 to 0.44 ± 0.43 (p < 0.001). No significant change of VA in controls.	Significant reduction of ME in TA-group (P < 0.001).
Chen et al.^108^	Case report.	1 eye (4 mg TA).	3 months.	Improvement in VA from counting fingers to 20/80.	Eye with macular ischaemia.
Chen et al.^109^	Prospective interventional non-controlled case series.	18 eyes (4 mg TA).	All patients completed 9 months, 12 eyes completed 12 months.	VA (logMAR) improved from 0.81 ± 0.36 to 0.65 ± 0.3 (p = 0.03) after 1 months, no significant difference in VA after 3, 6, 9 and 12 months.	Eye with macular ischaemia. All eyes with macular ischaemia.
Tsujikawa et al.^110^	Prospective interventional non-controlled case series.	17 eyes (vitrectomy + 10 mg TA intravitreal), 12 eyes of them with recurrent ME received sub-tenon 20 mg TA.	12.1 months.	82% of eyes rapid resolution of ME within 2 months (p = 0.041). 12 eyes (70.5%) received sub-tenon TA because of recurrent ME. Final VA (logMAR) improved from 0.74 ± 0.40 to 0.40 ± 0.34 (p = 0.010).	14 eyes with vitrectomy underwent additional phacoemulsification with lens implantation.
Karacorlu et al.^111^	Prospective interventional non-controlled case series.	8 eyes (4 mg TA). All eyes with serous macular detachment.	6 months.	After TA regression of ME and serous macular detachment in all eyes. After 6 months recurrence in 2 eyes (25%) re-treatment occurred. Final VA improved in 7 eyes (87.5%).	
Krepler et al.^112^	Prospective interventional non-controlled case series.	9 eyes (4 mg TA).	6 months.	Significant improvement in reading VA only after 1 month (p = 0.02). No significant improvement in VA for distance. No significant reduction in macular thickness.	5 eyes non-ischaemic BRVO. 4 eyes ischaemic BRVO.
Degenring et al.^113^	Case report.	2 patients: 1 eye BRVO 1 eye CRVO (25 mg TA).	5 weeks.	Patient with BRVO improved VA from 0.25 to 0.5. Patient with CRVO from 0.4 to 0.5.	
Wakabayshi et al.^114^	Prospective interventional non-controlled case series.	5 eyes with CRVO. 11 eyes with BRVO. All eyes received sub-tenon injection of 20 mg TA.	7 months.	8 eyes (50%) improved VA and 2 eyes (12.5%) had worsening of VA at the time of final examination. Reduction of ME >30% of initial thickness in 13 eyes (81.3%). Because of recurrent ME – in 7 eyes repeated sub-tenon application of TA.	1 eye with BRVO pretreated with laser photocoagulation because of retinal ischemia.
Salinas-Alaman et al.^115^	Retrospective interventional case series.	5 eyes (4 mg TA).	6 months	Improvement of VA in 4 eyes. 1 eye underwent re-injection after 3 months because of recurrent ME.	
Hirano et al.^116^	Retrospective interventional comparative case-control study.	8 eyes TA-injected group (simultaneous intravitreal and sub-tenon TA injection). 7 eyes vitrectomy with TA group (treated by vitrectomy and intravitreal or sub-tenon TA).	12 months.	VA improved significantly from baseline in both the TA-injected (p = 0.0069) and vitrectomy with TA groups (p = 0.0145). There was no significant difference in VA and macular thickness between the two groups.	
Kuppermann et al.^117^	Randomized interventional clinical trial.	105 (20 with venous occlusion) eyes in each group: I. 700 μg Posurdex II. 350 μg Posurdex III. controls.	3 months.	Improvement of VA of ≥10 lines (ETDRS) 35% eyes in group 700 μg Posurdex, 24% in 350 μg Posurdex and 13% in control group (p < 0.001 versus 700 μg group; p = 0.04 versus 350 μg group).	Preliminary report 60 eyes with retinal venous occlusion include eyes with CRVO and BRVO.

Most published studies on intravitreal TA for BRVO, however, suffer from two serious flaws: either the designs are not randomized or they often do not clearly differentiate between nonischemic types and ischemic types of occlusion. To compare the effectiveness and safety of standard care versus TA injection in the treatment of ME in patients with CRVO and BRVO, the multicenter randomized study SCORE is ongoing (https://web.emmes.com/study/score). In each of the two disease areas, 630 participants will be randomized in a 1:1:1 ratio to one of three groups: standard care, intravitreal 4 mg of TA, or 1 mg of TA. The follow-up is planned for 3 years. The results are not published as yet. Biodegradable intravitreal implants may allow steroid delivery over a more sustained period, permitting longer duration of action. A multicenter randomized clinical trial which evaluates implantation of dexamethasone 350 μg or 700 μg (Posurdex) versus observation (no therapy) for ME secondary to a variety of retinal disorders (including BRVO) has been reported.^117^ The preliminary 90-day results of all 315 evaluated patients showed that an improvement in VA of 10 letters or more (in ETDRS) was achieved by a greater proportion of patients treated with dexamethasone 700 μg (35%) or 350 μg (24%), than untreated patients (13%; *p* < 0.001 versus 700 μg group; *p* = 0.04 versus 350 μg group). The results were similar for patients with diabetic retinopathy, retinal vein occlusion, or uveitis or Irvine-Gass syndrome. In total, 60 patients with BRVO were randomized 1:1:1 to receive 350 μg or 700 μg dexamethasone or observation (no therapy). In the case of RVO, the effect of the treatment was evaluated only in a common group (CRVO and BRVO patients together): an improvement in VA of 10 letters or more was achieved in 15% of untreated patients versus 31% of patients treated with dexamethasone 700 μg. The number of patients with an increase in intraocular pressure of more than 10 mmHg from baseline anytime during the study was 12% for 350 μg, 17% for 700 μg, and 3% for the untreated controls.^117^

##### Periocular Application of Triamcinolone Acetonide

Kawaji et al.^122^ evaluated in 20 patients the effectiveness and safety of trans-tenon retrobulbar injection of 40 mg of TA for ME associated with BRVO after vitrectomy. Improvement in VA was seen in 14 (70%) eyes. Hayashi et al.^123^ compared in a randomized clinical trial, the short-term effect of intravitreal versus retrobulbar injection of TA for the treatment of ME caused by BRVO. Sixty patients received either a single intravitreal injection (4 mg) or repeated retrobulbar injections (40 mg, three times) of TA. The first injection in the retrobulbar group was given approximately one week after focal laser photocoagulation. Foveal thickness, macular volume, and improvement in VA were significantly better after intravitreal injection than after repeated retrobulbar injections. The need for re-injections was significantly greater in the retrobulbar group than in the intravitreal group.

#### Intravitreal Injection of VEGF Inhibitors

VEGF inhibitors are a treatment option for ME associated with RVO that target the disease at the causal molecular level. Randomized studies evaluating the results of treatment of all available VEGF inhibitors (bevacizumab, ranibizumab, and pegaptanib) are ongoing. Case reports, small retrospective or prospective non-controlled studies of VEGF inhibitors in the treatment of ME and retinal neovascularizations secondary to BRVO, have been published.^124^^–^140

Rosenfeld et al.^124^ first reported improved VA and reduced ME measured by optical coherent tomography (OCT) following intravitreal injection of bevacizumab for recurrent ME secondary to CRVO in an eye previously treated by intravitreal TA injection. In a short-term study, Iturralde et al.^125^ treated 16 eyes of CRVO with ME that had failed intravitreal corticosteroid therapy, and nearly every eye showed some anatomic or VA improvement following bevacizumab injection. In various reports, doses from 1.25 to 2.5 mg bevacizumab have been intravitreally administrated.^125^^–^134 The most recently published studies evaluated the results in a group of patients with BRVO combined with patients with CRVO. In all of these studies, bevacizumab injection improved VA and reduced macular thickness measured by OCT within the first 3 to 9 weeks. Few studies are available for BRVO patients alone.^126^^,^^127^ Rabena et al.^126^ reported a significantly increased VA and reduced macular thickness after treatment with 1.25 mg bevacizumab in a retrospective study of 27 patients with BRVO. Recurrent ME was observed in 6 (22%) patients an average of 2.1 months after the initial injection. These patients were reinjected and all showed moderate to complete reduction in ME. The limitations of this retrospective study are short follow-up and lack of control group. Additionally, most of the eyes in the study were previously treated and thus failed standard treatment, and perhaps represent a group unlikely to benefit from any treatment. All published reports provide evidence that this treatment is well tolerated. The most common adverse events were conjunctival hyperemia and subconjunctival hemorrhage at the injection site. However, the duration of reduced ME after bevacizumab administration is currently unknown. Frequent repeated injections are required to prevent a rebound effect with no clearly defined endpoint.^128^

Campochiaro et al.^129^ presented preliminary results of a randomized study in the treatment of BRVO with intravitreal injection of ranibizumab at the 2007 Annual Meeting of Association on Research and Vision in Ophthalmology (ARVO). Patients with ME due to CRVO or BRVO were randomized 1:1 to receive 3 monthly injections of 0.5 or 0.3 mg of ranibizumab. Interim results without regard to treatment assignment, which is unknown, showed that 12 randomized patients with BRVO gained an improvement in VA (in ETDRS) from 21 to 37 letters and a reduction in ME from 508 to 208 μm after 3 months of treatment. The endpoint results are expected to clarify any differences between the treatment groups. Another indication for anti-VEGF drugs are retinal neovascularizations, rubeosis iridis, and neovascular glaucoma. Rapid regression of neovascularizations and compensation of intraocular pressure have been described in several studies.^136^^–^139 Intracameral application of bevacizumab as successful treatment of rubeosis iridis and neovascular glaucoma has also been reported.^140^

Prospective, controlled studies are mandatory to develop standardized treatment protocols that allow safe and effective application of anti-VEGF drugs.

#### Laser Treatment

Laser treatment is an established method for use in patients with BRVO. A large number of publications concerning the role of photocoagulation in the management of BRVO have appeared. Various laser techniques can be used: macula grid photocoagulation and the method of arterial crimping for treatment of ME, and peripheral scatter photocoagulation for treatment of retinal and/or disc neovascularization.

##### Macular Grid Laser Photocoagulation

The Branch vein occlusion study group remains the largest randomized prospective trial that has evaluated the efficacy of grid-pattern laser photocoagulation for the treatment of ME in BRVO.^141^ In this study, only eyes with recent BRVO, perfused ME, resolved foveal hemorrhage, VA 20/40 or worse, and no other ocular comorbidities were included. After a 3-year follow-up period, 65% of treated eyes gained improvement of 2 or more lines from baseline, as opposed to 37% of untreated eyes. The number of eyes that lost 2 or more lines was not significantly different in the two groups.^141^ Parodi et al. published two randomized controlled studies, in which no significant benefit of MLG on VA was found.^142^^,^^143^ MLG is recommended as an effective treatment to reduce the ME in BRVO after a period of 3 to 6 months after onset and following absorption of the majority of hemorrhage if VA is 20/40 or worse.^61^^,^^62^^,^^141^ If the fluorescein angiogram reveals macular nonperfusion, laser therapy is not warranted.^141^ Subramanian et al.^65^ recommended laser treatment in patients with poor VA (20/200 or worse) secondary to ME due to BRVO, before more aggressive approaches (as intravitreal TA). Argon MLG is usually used for this purpose. However, diode laser (810 nm) and krypton red laser (647 nm) also can be used.^63^^,^^141^^,^^144^

##### Scatter Photocoagulation

The randomized controlled study by Branch vein occlusion study group^54^ reported that peripheral scatter laser photocoagulation significantly reduced the development of retinal neovascularization and vitreous hemorrhage. This study also demonstrated that, if all eyes with large retinal nonperfusion were treated, 64% of these patients would never develop neovascularization. If only the eyes that develop neovascularization were treated, the events of vitreous hemorrhage would decrease from 61% to 29%. Since loss in the lower part of the visual field can produce marked disability and BRVO involving the superior retina is common, a significant worsening of visual fields with laser treatment becomes a very important, clinically relevant finding.^145^ Therefore, waiting is generally advocated until neovascularization actually develops before scatter photocoagulation is considered.^54^

##### Arteriolar Constriction

An alternative type of laser treatment involves arteriolar constriction (called also “crimping technique”) and may be considered in order to reduce the inflow into the affected area if the ME is excessive. This procedure was first described by L'Esperance^146^ in 1975. It may lead to a decrease in arterial pressure in the occluded region resulting in better drainage of the ME due to reduced blood inflow. The technique is employed by placing coagulations at approximate intervals of ½ disc diameter (using the green beam of argon laser) through the afferent arteriole in the region of venous blockage. In 1984 Jalkh et al.^64^ proposed their own modification of this method and published the results obtained in 41 eyes. In this study, arterial constriction was applied in the treatment of the chronic stage of BRVO. Rehak et al. published several studies describing the modified arteriolar constriction in patients with BRVO.^147^^–^149 This technique consists of the application of coalescent coagulation spots through the afferent arteriole that supports the occluded venous region. 83% of patients treated by this method within the first 2 months after the onset of occlusion achieved a final VA 20/40 or better.^149^ In a study by Erdol and Akyol,^150^ the improvement in VA was higher in a group of patients receiving the MLG combined with arteriolar constriction than in a group treated by MLG only. However, the difference in the resolution of ME between the groups was not statistically significant. The authors suggest that arteriolar constriction in addition to grid pattern laser photocoagulation is more effective for resolving ME in patients with BRVO.

## CONCLUSIONS

The pathogenesis of BRVO is multifactorial. Its resulting visual loss is due primarily to ME, macular nonperfusion, and retinal neovascularization. A large number of treatments have been advocated in its management. Unfortunately, almost all of these lack sufficient evidence for their effectiveness. Randomized prospective trials are essential. The only one established treatment for ME is macular grid photocoagulation in patients with BRVO longer than 3 months and a VA of 20/40 or worse. Additionally, the initial VA may play a crucial role in the prognosis of BRVO and determinates the final VA.
